# Aptamer-Adjusted Carbon Dot Catalysis-Silver Nanosol SERS Spectrometry for Bisphenol A Detection

**DOI:** 10.3390/nano12081374

**Published:** 2022-04-17

**Authors:** Yuqi Xie, Lu Ma, Shaoming Ling, Huixiang Ouyang, Aihui Liang, Zhiliang Jiang

**Affiliations:** 1 Key Laboratory of Regional Ecological Environment Analysis and Pollution Control in Western Guangxi (Baise University), Education Department of Guangxi Zhuang Autonomous Region, College of Chemistry and Environment Engineering, Baise University, Baise 533000, China; xiekey@sina.com (Y.X.); malulu2022@163.com (L.M.); lingshaoming@sohu.com (S.L.); 2 Guangxi Key Laboratory of Environmental Pollution Control Theory and Technology, Guangxi Normal University, Guilin 541004, China; ahliang2008@163.com

**Keywords:** CDs catalysis, aptamer adjust, SERS, BPA

## Abstract

Carbon dots (CDs) can be prepared from various organic (abundant) compounds that are rich in surfaces with –OH, –COOH, and –NH_2_ groups. Therefore, CDs exhibit good biocompatibility and electron transfer ability, allowing flexible surface modification and accelerated electron transfer during catalysis. Herein, CDs were prepared using a hydrothermal method with fructose, saccharose, and citric acid as C sources and urea as an N dopant. The as-prepared CDs were used to catalyze AgNO_3_–trisodium citrate (TSC) to produce Ag nanoparticles (AgNPs). The surface-enhanced Raman scattering (SERS) intensity increased with the increasing CDs concentration with Victoria blue B (VBB) as a signal molecule. The CDs exhibited a strong catalytic activity, with the highest activity shown by fructose-based CDs. After N doping, catalytic performance improved; with the passivation of a wrapped aptamer, the electron transfer was effectively disrupted (retarded). This resulted in the inhibition of the reaction and a decrease in the SERS intensity. When bisphenol A (BPA) was added, it specifically bound to the aptamer and CDs were released, recovering catalytical activity. The SERS intensity increased with BPA over the concentration range of 0.33–66.67 nmol/L. Thus, the aptamer-adjusted nanocatalytic SERS method can be applied for BPA detection.

## 1. Introduction

Because of their high selectivity, high affinity, and low concentration dissociation, aptamer (Apt) reactions have been widely used in biomedicine, analytical chemistry, and clinical examination [1,2,3,4,5,6,7,8]. Through surface-enhanced Raman scattering (SERS), an enhanced Raman signal is obtained for the molecules adsorbed on or close to the metal surface and activated by its local surface plasma resonance (LSPR) [9,10,11,12,13]. With the development of nanoparticle preparation technology, SERS substrates have become more flexible and inexpensive, allowing them to be modified and fixed on slides or optical fibers [14,15] or used directly in colloids [16]. These advantages have resulted in the widespread use of SERS nanosubstrates with local surface plasmon effects [17,18,19,20]. In addition, nanocatalysis has been conducted to generate noble metal nanoparticles. The generated nanoparticles have been subsequently used as a direct SERS substrate based on the LSPR effect and subjected to the Apt reaction to establish an analysis platform [21,22,23].

Carbon dots (CDs) typically exhibit good biocompatibility and have been widely used as bioimaging probes and biosensors [24,25]. CDs have different preparation methods and numerous sources (including carbohydrates, amino acids, and organic acids) that promote flexible structure modification [26,27,28,29]. In addition, CDs exhibit good electronic transfer ability and can be used to catalyze redox reactions [30,31,32,33] and to establish analysis methods. Long groups [31] prepared CDs with lampblack followed by reduction with NaBH_4_ to form r-CDs, which was then used to catalyze the reaction of hydrogen peroxide with 3,3′,5,5′-tetramethylbenzidine for hydrogen peroxide detection (detection range: 0.01–0.1 mM). Wang and coworkers [32] synthesized CDs containing O, N, Fe, S, and C by the hydrothermal treatment of animal blood. The as-prepared CDs exhibited excellent peroxidase-like catalytic activity and could be mixed with glucolase to determine glucose by colorimetry with a detection range of 0.2–2.5 mM. However, the combination of Apt-adjusted CD catalysis and Ag nanosol SERS for the detection of bisphenol A (BPA), which is an endocrine disruptor, has not been extensively investigated.

Endocrine-disrupting chemicals (EDCs) can damage human and ecosystem health by inhibiting reproduction as well as causing birth defects, dysplasia, and metabolic disorders. Prolonged exposure to EDCs may cause obesity, diabetes, cardiovascular disease, carcinogenesis, and neurotoxicity [34]. BPA is a common industrial material that has been detected as a potential risk for human diseases, as evaluated by supervision organizations and health agencies [35,36,37]. BPA threatens health through food, when it is present in a container during food heating [38]. The main methods of detecting BPA include chromatography [39,40], absorption spectroscopy [41,42], fluorimetry [43,44], electrochemistry methods [45], resonance Rayleigh scattering (RRS) [46,47], and surface-enhance Raman spectroscopy [48,49]. However, these methods exhibit low selectivity and low sensitivity or require precious and expensive instrumentation. Thus, real-time detection of BPA is difficult. Herein, an SERS method for BPA detection was developed using as-prepared Ag nanoparticles (AgNPs) with a strong surface plasmon resonance effect as an SERS substrate.

## 2. Results and Discussion

### 2.1. Principle

**Figure 1:**
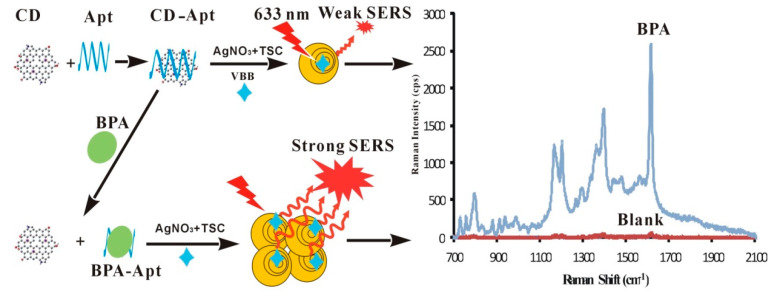
Mechanism of the carbon dots (CDs) catalytic reaction and the analysis principle.

The carbon dots (CDs) surface contains abundant electrons, which can accelerate the electron transfer between the oxidant and the reductant, allowing the redox reaction to proceed more easily. At a certain concentration of silver nitrate (AgNO_3_)–trisodium citrate (TSC), effective collisions occur infrequently between citrate and silver ions. When CDs are added, they adsorb silver ions and citrate molecules on the surface and rapidly transfer electrons from citrate to silver ions, resulting in the generation of elemental silver, 1,3-acetonedicarboxylic acid, and CO_2_. The as-prepared Ag nanoparticles (AgNPs) increased with increasing CDs loading (Figure 1). The SERS signal of the system was strong with AgNPs as the SERS substrate and Victoria blue B (VBB) as the molecular probe. When an aptamer (Apt) enwrapped the surface of the CDs, the absorption of citrate and silver ions on the CDs was blocked, inhibiting the catalytic activity. This resulted in a decrease in the SERS intensity. In the presence of BPA, a specific bonding with the Apt was achieved, resulting in CDs exposed to the reaction system and recovering the catalytic activity. The generated AgNPs increased with BPA loading, and the SERS signals were linearly increased, allowing for the development of an SERS method for BPA detection.

### 2.2. SERS Spectra

**Figure 2:**
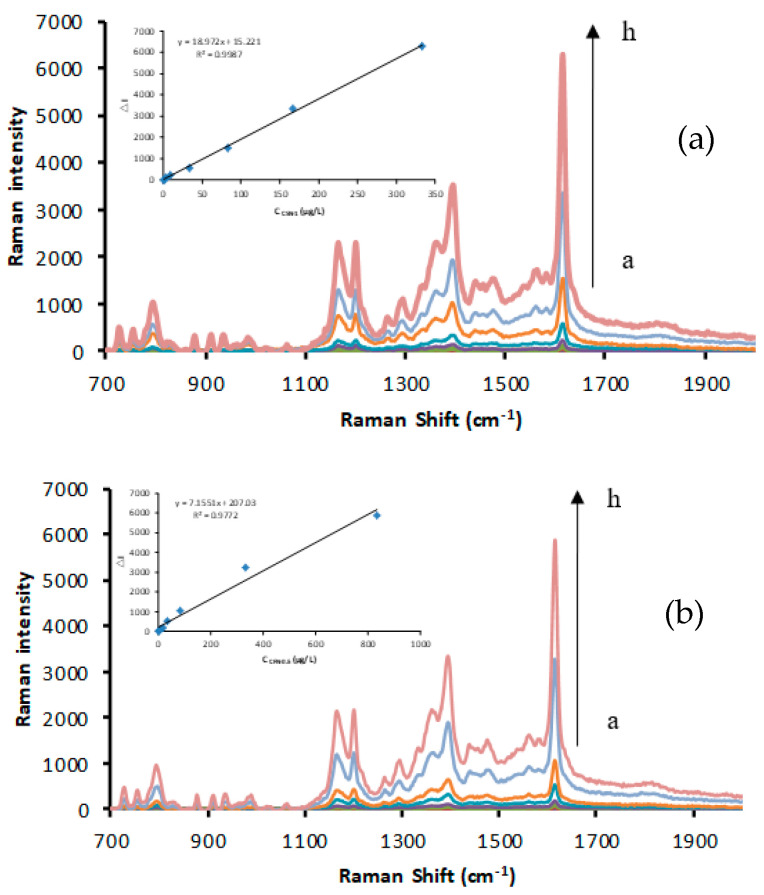
Surface−enhanced Raman scattering (SERS) spectra. (**a**) a to h: solutions of CD−FN3 (0, 1.67, 3.33, 8.33, 33.33, 83.33, 166.67, and 333.33 μg/L) + 1.33 mmol/L AgNO_3_ + 4.67 mmol/L TSC + 3.33 × 10^−7^ mol/L Victoria blue B (VBB) + 0.02 mol/L NaCl; (**b**) a to h: solutions of CD−SN2 (0, 3.33, 8.33, 16.67, 33.33, 83.33, 333.33, and 833.33 μg/L) + 1.33 mmol/L AgNO_3_ + 4.67 mmol/L TSC +3.33 × 10^−7^ mol/L VBB + 0.02 mol/L NaCl.

**Figure 3:**
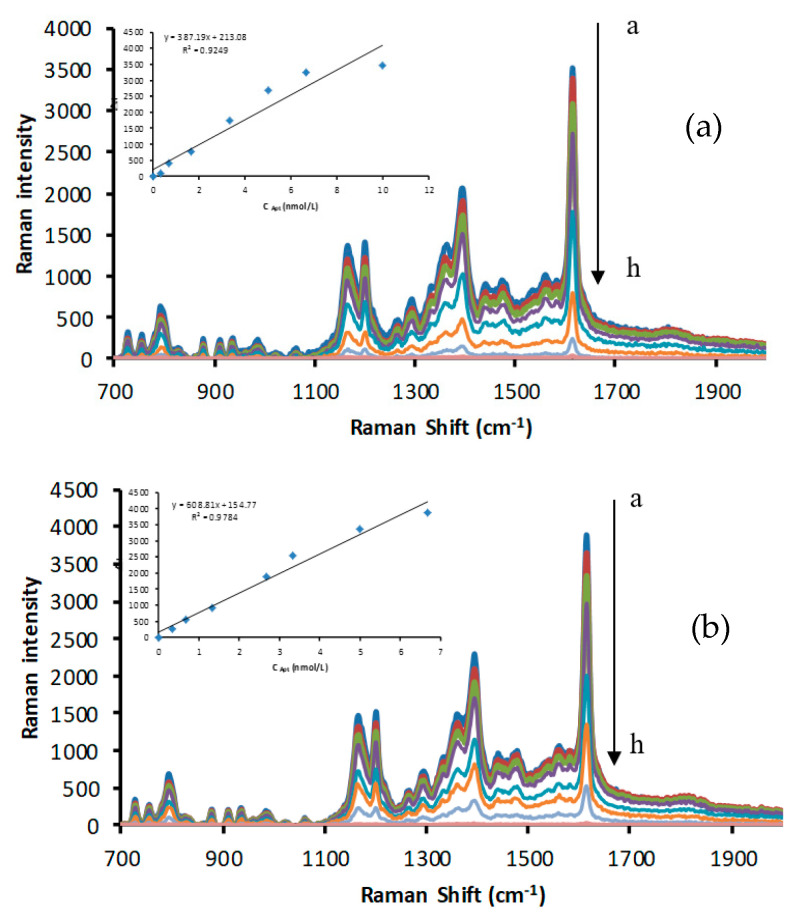
SERS spectra of aptamer (Apt)−CD−AgNO_3_−TSC: (**a**) a to h: solutions of Apt (0, 0.33, 0.67, 1.67, 3.33, 5, 6.67, and 10 nmol/L) + 166.67 μg/L CD−FN3 + 1.33 mmol/L AgNO_3_ + 4.67 mmol/L TSC +3.33 × 10^−7^ mol/L VBB + 0.02 mol/L NaCl; (**b**) a to h: solutions of Apt (0, 0.33, 0.67, 1.33, 2.67, 3.33, 5, and 6.67 nmol/L) + 333.33 μg/L CD−SN2 + 1.33 mmol/L AgNO_3_ + 4.67 mmol/L TSC+3.33 × 10^−7^ mol/L VBB + 0.02 mol/L NaCl.

**Figure 4:**
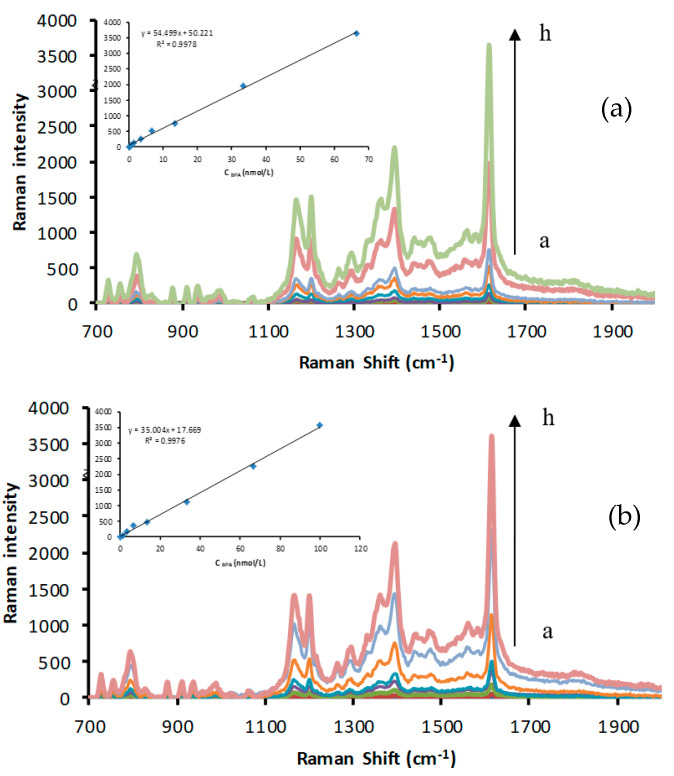
SERS spectra of bisphenol A (BPA)−Apt−CD−AgNO_3_−TSC: (**a**) a to h: solutions of 3.33 nmol/L Apt + 166.67 μg/L CD−FN3 + BPA (0, 0.33, 0.67, 1.33, 3.33, 6.67, 13.33, 33.33, and 66.67 nmol/L) + 1.33 mmol/L AgNO_3_ + 4.67 mmol/L TSC+3.33 × 10^−7^ mol/L VBB + 0.02 mol/L NaCl; (**b**) a to h: solutions of6.67 nmol/L Apt + 333.33 μg/L CD−SN2 + BPA (0, 1.33, 3.33, 6.67, 13.33, 33.33, 66.67, and 100 nmol/L) + 1.33 mmol/L AgNO_3_ + 4.67 mmol/L TSC + 3.33 × 10^−7^ mol/L VBB + 0.02 mol/L NaCl.

At 85 °C, the reaction of silver nitrate–TSC was blocked, but in the presence of CDs, the redox reaction proceeded to produce yellow AgNPs. When VBB was used as a signal molecule, four enhanced SERS peaks were observed at 1614, 1394, 1200, and 795 cm^−1^. The 795 cm^−1^ peak can be attributed to the inner surface deformation of the ring. The 1200 cm^−1^ peak was attributed to the outside surface deformation of NH_2_, while the 1394 cm^−1^ peak was assigned to C–H of C=C and C–H bending vibration. The 1614 cm^−1^ peak was assigned to the C=C and C=N stretching vibration of the benzene ring [50]. In addition, the 1614 cm^−1^ peak was the most intense and linearly increased with the increasing CD concentration. Various C sources were selected to prepare CDs (glucose, fructose, sucrose, and citric acid), and urea was used as an N source to prepare N-CDs for catalysis investigation (Figure 2 and Figures S1–S3). In the presence of the Apt, the CD surfaces were enwrapped and isolated from the catalytic system, suppressing the CD catalytic activity and the SERS intensity (Figure 3 and Figure S4). When BPA was added, it specifically conjugated with the Apt, releasing CDs and recovering catalytic activity. With the increasing BPA loading, the amount of released CDs increased, and the generated AgNPs increased with the SERS intensity as a function of BPA content (Figure 4 and Figures S5–S7).

### 2.3. The Catalytic Effect of CDs and the Inhibition of the Apt

Under the optimal conditions, AgNO_3_ slowly reacted with TSC. When the nanocatalyst was added, the small particle size, high surface energy, and high surface electron density allowed silver ions and citrate to absorb on its surface, facilitating electron transfer. The reaction generated yellow AgNPs, and the system SERS intensity increased rapidly due to the accelerated electron transfer. The catalytic activities of various catalysts, i.e., the as-prepared CDs using glucose, fructose, sucrose, citric acid, and the corresponding N dopants, as well as AgNPs, were investigated. The CDs produced with pure citric acid as a C source showed no catalysis, while the others had strong catalysis with N doping further increasing the catalytic activity (Table 1). This indicated that N atoms in the CD crystal lattice facilitated the incorporation between CDs and –COOH or –NH_2_ by non-covalent hydrogen bonds and Van der Waals forces. The as-prepared AgNPs caused the SERS value to increased (Table S1). In addition, AgNPs could catalyze this reaction even at the concentration of 13.33 nmol/L, indicating that the as-prepared AgNPs were autocatalytic (Figure S8).

### 2.4. Scanning Electron Microscopy (SEM)

**Figure 5:**
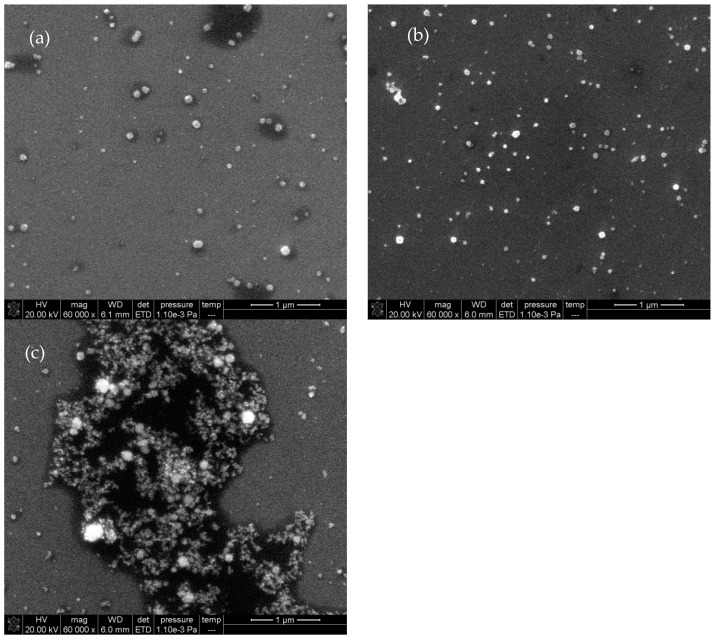
SEM images of Apt–CD-FN3–AgNO_3_–TSC–BPA system (20.67 nmol/L Apt + 333.33 μg/L CD-FN3 + 1.33 mmol/L AgNO_3_ + 4.67 mmol/L TSC) at the temperature of 85 °C for 21 min with different BPA concentrations: (**a**) 0 nmol/L; (**b**) 3.33 nmol/L; (**c**) 13.33 nmol/L.

The reaction solution was diluted up to 10 times for final BPA concentrations of 0, 3.33, and 13.33 nmol/L. Subsequently, a 10 μL sample solution was dropped onto a silicon wafer and dried naturally before conducting SEM. As can be seen in Figure 5a, in the absence of BPA, few AgNPs with a mean grain size of 100 nm were present in the reaction solution. Upon BPA addition, the catalytic activity was recovered, and AgNPs were formed by aggregation, with a mean grain size of 70 nm (Figure 5b,c), as corroborated by the laser scattering image (Figure S9).

### 2.5. Optimization of Catalysis Conditions

The effect of the reagent concentration on the determination was studied. With the increasing AgNO_3_ concentration, the amount of generated AgNPs increased with the SERS value, while the △I value was largest at the AgNO_3_ concentration of 1.33 mmol/L (Figure S10). When the AgNO_3_ concentration increased continuously, the SERS value decreased conversely, because the AgNPs aggregated excessively and the control test also reacted. Therefore, 1.33 mmol/L AgNO_3_ was chosen for subsequent use. With the increasing TSC concentration, the amount of generated AgNPs increased, the SERS value increased, and the △I value was maximized at the TSC concentration of 4.67 mmol/L (Figure S11). Thus, 4.67 mmol/L TSC was selected as optimal. The reaction temperature significantly influenced the generated AgNPs, and at 85 °C for 21 min, the △I value was maximized. Thus, 85 °C and 21 min were chosen as the optimal conditions for the reaction (Figures S12 and S13). The effect of Apts was also studied, and the △I value reached the maximum at the Apt concentration of 13.33 nmol/L (Figure S14). In this examination, some time was required for the combination of the Apt with fullerol. With the increasing of the binding time, the combination strengthened within 8 min (Figure S15). With the increasing time, the △I value was maintained; thus, to ensure sufficient stability, a binding time of 10 min was selected as optimal.

### 2.6. Working Curve

**Figure 6:**
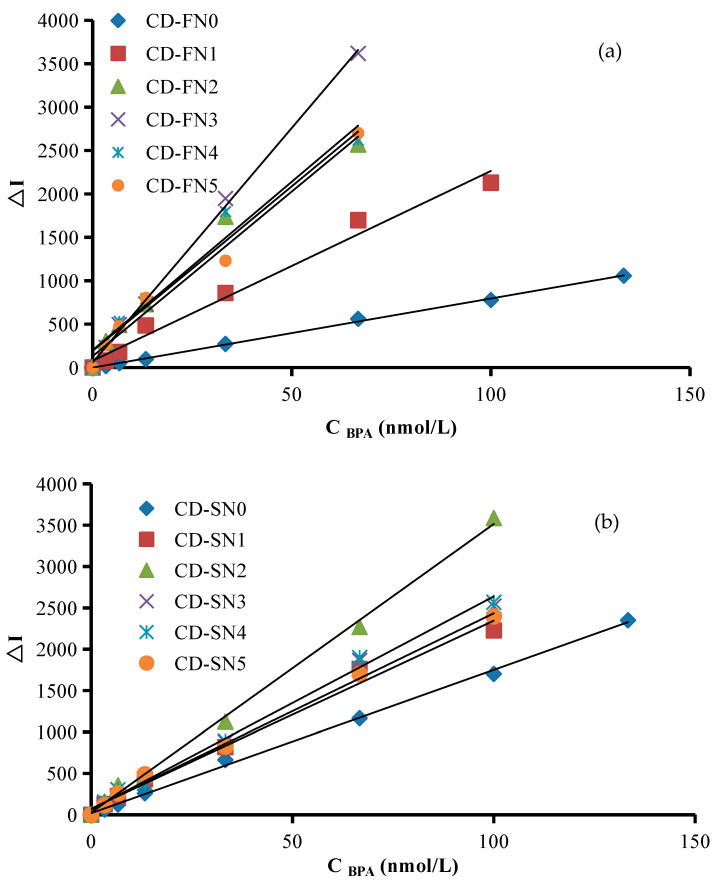
Working curves for the SERS determination of Apt–CD–AgNO_3_–TSC–BPA: (**a**) the solution of 6.67 nmol/L Apt + 3.33–133.33 nmol/L BPA + CD-FN + 1.33 mmol/L AgNO_3_ + 4.67 mmol/L TSC + 3.33 × 10^−7^ mol/L VBB + 0.02 mol/L NaCl; (**b**) the solution of 10.00 nmol/L Apt + 3.33–133.33 nmol/L BPA + CD-SN + 1.33 mmol/L AgNO_3_ + 4.67 mmol/L TSC + 3.33 × 10^−7^ mol/L VBB + 0.02 mol/L NaCl.

Under the optimal conditions, the working curves were prepared according to the relationship between C_(__BPA)_ and the corresponding ΔI_1614 cm_^−1^ values (Figure 6 and Figures S16–S19), and the analytical characteristics are listed in Table 1. The SERS method showed the maximum slope of 54.50 with a limit of detection of 0.1 nmol/L. These methods were compared with previously reported methods for BPA determination. The newly developed method was simple and showed high sensitivity and good selectivity. Therefore, it can be used to detect residues BPA in plastic products.

### 2.7. Influence of Substances

According to the procedure, CD-FN was used as a catalyst, and the influence of the coexisting interfering substances on the determination of 3.33 nmol/L BPA was tested. The common substances tested did not interfere with the determination with a relative error of ±10% (Table S2).

### 2.8. Sample Analysis

Different brands of plastic films and polythene bags, unbranded grocery bag, and 2 disposable plastic drinking cup brands were purchased from the market and snipped. Then, 0.4 g of the samples was soaked for 48 h in ethyl alcohol. The extracts were then air-dried in a well-ventilated area, subsequently dissolved in 100 mL of double-distilled water and stored at 4 °C. According to the procedure, 50 μL of the samples were used to detect BPA. A known amount of BPA was added to the sample, and recoveries of 98.5–105.4% were obtained (Table S3). 

## 3. Materials and Methods

### 3.1. Apparatus

A model DXR smart Raman spectrometer (Thermo Company, Waltham, MA, USA) with a laser wavelength of 633 nm and a laser power of 3 mW, a Cary Eclipse fluorescence spectrophotometer (Varian Company, Palo Alto, CA, USA), a TU-1901 double-beam UV-visible spectrophotometer (Beijing General Instrument Co., LTD, Beijing, China), and an FEI Quanta 200 FEG field-emission scanning electron microscope (FEI Company, Hillsboro, OR, USA) were used.

### 3.2. Reagents

Apt with a sequence of 5′-3′ GGG CCG TTC GAA CAC GAG CAT G N_60_ GG ACA GTA CTC AGG TCA TCC TAG G (Sangon Biotech (Shanghai) Co., Ltd., China). 1.0 × 10^−3^ mol/L BPA: 22.8 mg BPA were dissolved with 2.0 mL ethanol and then diluted to 100 mL with water (0.1 mol/L, measured by the HPLC method [51]). The solution was diluted and used step by step. 0.01 mol/L silver nitrate (Sinopharm chemical reagent Co. Ltd., China); 0.1 mol/L TSC (Xilong Scientific Co., Ltd., Shantou, China); 10^−3^ mol/L VBB solution: 25 mg VBB were dissolved with 5.0 mL ethanol and then diluted to 50 mL with water. The solution was diluted and used step by step. glucose; fructose; sucrose; citric acid; urea; and Ca(OH)_2_ (Sinopharm chemical reagent Co. Ltd., Shanghai, China). All reagents were analytically pure, and water was double-distilled.

Preparation of N-CDs (CD-GN): 1 g glucose and 1 g urea ultrasonic dissolved in 30 mL water (N: 11.6%) to form a yellow solution. The mixture was transferred into a high-pressure reaction kettle heated with polytetrafluoroethylene lining for 180 °C for 5 h and then air-cooled to room temperature. The reaction mixture was a brown yellow solution. Then, it was dialyzed for 12 h with an MWCO 3500Da dialysis bag, and the water was changed at every 2 h until the dialysate was colorless. The CDs were adjusted to neutral with 50 mmol/L NaOH and then diluted to 30 mL with water. The CD_GN_ concentration was 0.025 g/mL.

Preparation of N-CDs (CD-FN): 1 g fructose and urea (0, 0.2, 0.5, 1.0, 1.5, and 2.0 g) ultrasonically dissolved in 30 mL water to form a yellow solution, marked as CD-FN0, CD-FN1, CD-FN2, CD-FN3, CD-FN4, and CD-FN5, respectively. The mixture was transferred into a high-pressure reaction kettle heated with polytetrafluoroethylene lining at 180 °C for 5 h and then air-cooled to room temperature. The reaction mixture was a brown yellow solution. Then, it was dialyzed for 12 h with an MWCO 3500Da dialysis bag, and the water was changed at every 2 h until the dialysate was colorless. The CDs were adjusted to neutral with 50 mmol/L NaOH and then diluted to 30 mL with water. The CD-FN concentration was 0.025 g/mL.

Preparation of N-CDs (CD-SN): 1 g sucrose and urea (0, 0.2, 0.5, 1.0, 1.5, 2.0 g) ultrasonically dissolved in 30 mL water to form a yellow solution, marked as CD-SN0, CD-SN1, CD-SN2, CD-SN3, CD-SN4, and CD-SN5, respectively. The mixture was transferred into a high-pressure reaction kettle heated with polytetrafluoroethylene lining at 180 °C for 5 h and then air-cooled to room temperature. The reaction mixture was a brown yellow solution. Then, it was dialyzed for 12 h with an MWCO 3500Da dialysis bag, and the water was changed at every 2 h until the dialysate was colorless. The CDs were adjusted to neutral with 50 mmol/L NaOH and then diluted to 30 mL with water. The CD-SN concentration was 0.025 g/mL.

Preparation of Ca-CDs (CD_Ca_): 1 g citric acid and 0.4 g Ca(OH)_2_ were dissolved in a reaction kettle with 10 mL water, and then, 500 μL ethidene diamine were added slowly and mixed well. The mixture was heated at 200 °C for 4 h in a muffle furnace. Then, the reaction mixture was centrifuged at 10,000 rad/s for 10 min. The precipitate dissolved in water and adjusted to pH 7.5 with 50 mmol/L NaOH and then diluted to 10 mL with water. The CD_Ca_ concentration was 0.1 g/mL.

Preparation of N-CDs (CD-CN): 1 g citric acid and urea (0, 0.2, 0.5, 1.0, 1.5, 2.0 g) ultrasonically dissolved in 30 mL water to form a yellow solution, marked as CD-CN0, CD-CN1, CD-CN2, CD-CN3, CD-CN4, and CD-CN5, respectively. The mixture was transferred into a high-pressure reaction kettle heated with polytetrafluoroethylene lining at 180 °C for 5 h and then air-cooled to room temperature. The reaction mixture was a brown yellow solution. Then, it was dialyzed for 12 h with an MWCO 3500Da dialysis bag, and the water was changed at every 2 h until the dialysate was colorless. The CDs solution was adjusted to neutral with 50 mmol/L NaOH and then diluted to 30 mL with water. The CD-CN was 0.025 g/mL.

### 3.3. Procedure

First, 20 µL of 1.5 µmol/L Apt, a certain amount of BPA, and 15 µL of a 0.02 g/L CD solution were added to a 5 mL graduated tube, mixed well and reacted for 20 min. Then, 200 µL of 0.01 mol/L AgNO_3_ and 70 µL of 0.1 mol/L TSC were added, and the mixture was diluted to 1.5 mL with water. The mixture was subsequently heated for 21 min in an 85 °C water bath and cooled with ice water. Next, 50 μL of 1.0 × 10^−5^ mol/L VBB and 40 μL of 1 mol/L NaCl were added and mixed well. The SERS spectra were recorded using a Raman spectrometer. The reaction solution SERS intensity at 1614 cm^−1^ (I_1614 cm_^−1^) and that of a blank solution without BPA (I_0_) were recorded, allowing the value of △I = $I_{1614{\;\mathrm{cm}}^{-1}}$ −I_0_ to be calculated.

## 4. Conclusions

The prepared CDs had a high surface effect and effectively catalyzed the reaction of TSC and silver nitrate to produce yellow AgNPs. The generated AgNPs showed strong SERS effects, with the SERS intensity linearly increased with the CD loading. When CDs were enwrapped using an Apt, the CD–silver ion binding was blocked, suppressing the catalytic activity. BPA specifically conjugated with the Apt, releasing the CDs and recovering the catalytic activity. The system SERS intensity linearly increased with the increasing BPA content. Therefore, an Apt-adjusted nanocatalysis and an SPR effect spectral analysis for BPA detection were established with excellent sensitivity, selectivity, simplicity, and rapidness.

## Figures and Tables

**Table 1:** Analytical characteristics of the Apt-adjusted catalysis and Ag nanoplasma SERS for the determination of BPA.

Test Method	Nanocatalyst	Working Curve	Linearly Range	Coefficient (R^2^)	Limit of Detection
SERS	CD-FN0	$\Delta I_{1614{\;\mathrm{cm}}^{-1}}$ = 7.96 C − 0.81	3.33–133.33 nmol/L	0.9988	1.2 nmol/L
	CD-FN1	$\Delta I_{1614{\;\mathrm{cm}}^{-1}}$= 22.12 C + 59.70	1.33–100 nmol/L	0.983	0.8 nmol/L
	CD-FN2	$\Delta I_{1614{\;\mathrm{cm}}^{-1}}$ = 39.16 C + 132.51	0.67–66.67 nmol/L	0.9709	0.2 nmol/L
	CD-FN3	$\Delta I_{1614{\;\mathrm{cm}}^{-1}}$ = 54.50 C + 50.22	0.33–66.67 nmol/L	0.9978	0.1 nmol/L
	CD-FN4	$\Delta I_{1614{\;\mathrm{cm}}^{-1}}$ = 40.13 C + 136.5	0.67–66.67 nmol/L	0.966	0.3 nmol/L
	CD-FN5	$\Delta I_{1614{\;\mathrm{cm}}^{-1}}$ = 38.49 C + 104.61	0.67–66.67 nmol/L	0.984	0.3 nmol/L
	CD-SN0	$\Delta I_{1614{\;\mathrm{cm}}^{-1}}$ = 17.31 C + 18.75	3.33–133.33 nmol/L	0.9984	2.0 nmol/L
	CD-SN1	$\Delta I_{1614{\;\mathrm{cm}}^{-1}}$ =22.93 C + 58.28	1.33–100 nmol/L	0.9886	0.8 nmol/L
	CD-SN2	$\Delta I_{1614{\;\mathrm{cm}}^{-1}}$ = 35.00 C + 17.67	1.33–100 nmol/L	0.9976	0.5 nmol/L
	CD-SN3	$\Delta I_{1614{\;\mathrm{cm}}^{-1}}$ = 25.67 C + 32.60	1.33–100 nmol/L	0.996	0.6 nmol/L
	CD-SN4	$\Delta I_{1614{\;\mathrm{cm}}^{-1}}$ = 25.98 C + 46.79	1.33–100 nmol/L	0.9946	0.7 nmol/L
	CD-SN5	$\Delta I_{1614{\;\mathrm{cm}}^{-1}}$ = 23.81 C + 57.91	1.33–100 nmol/L	0.9949	0.7 nmol/L
	CD	$\Delta I_{1614{\;\mathrm{cm}}^{-1}}$ = 34.12 C + 46.86	0.67–66.67 nmol/L	0.9896	0.3 nmol/L
	CD_GN_	$\Delta I_{1614{\;\mathrm{cm}}^{-1}}$ = 15.07 C + 45.55	0.67–66.67 nmol/L	0.9759	0.5 nmol/L
	CD_Ca_	$\Delta I_{1614{\;\mathrm{cm}}^{-1}}$ = 19.09 C + 47.84	0.67–66.67 nmol/L	0.9851	0.45 nmol/L
	CD-CN1	$\Delta I_{1614{\;\mathrm{cm}}^{-1}}$ = 8.66 C − 0.48	1.33–100 nmol/L	0.997	0.7 nmol/L
	CD-CN2	$\Delta I_{1614{\;\mathrm{cm}}^{-1}}$ = 31.47 C + 56.14	0.67–66.67 nmol/L	0.9938	0.3 nmol/L
	CD-CN3	$\Delta I_{1614{\;\mathrm{cm}}^{-1}}$ = 25.21 C + 45.16	0.67–66.67 nmol/L	0.9889	0.4 nmol/L
	CD-CN4	$\Delta I_{1614{\;\mathrm{cm}}^{-1}}$ = 21.85 C + 41.74	0.67–66.67 nmol/L	0.9928	0.4 nmol/L
	CD-CN5	$\Delta I_{1614{\;\mathrm{cm}}^{-1}}$ = 18.39 C + 44.54	0.67–66.67 nmol/L	0.9812	0.5 nmol/L
	Ag nanoparticle (AgNP)	$\Delta I_{1614{\;\mathrm{cm}}^{-1}}$ = 27.24 C + 38.92	0.67–66.67 nmol/L	0.9938	0.3 nmol/L

## Data Availability

Not applicable.

## Supplementary Materials

The following are available online at https://www.mdpi.com/article/10.3390/nano12081374/s1, Figure S1: SERS spectra of CD_GN_–AgNO_3_–TSC, Figure S2: SERS spectra of CD_Ca_–AgNO_3_-TSC, Figure S3: SERS spectra of CD-CN–AgNO_3_–TSC, Figure S4 SERS spectra of Apt–CD-CN–AgNO_3_–TSC, Figure S5: SERS spectra of BPA–Apt–CD-GN–AgNO_3_–TSC, Figure S6: SERS spectra of BPA–Apt–CD_Ca_–AgNO_3_–TSC, Figure S7: SERS spectra of BPA–Apt–CD-CN–AgNO_3_–TSC, Figure S8: The effect of AgNPs on the SERS intensity, Figure S9: Laser scattering image of Apt–CD-FN–AgNO_3_–TSC–BPA system, Figure S10: Effect of AgNO_3_ concentration on the △I value, Figure S11: Effect of TSC concentration on the △I value, Figure S12: Effect of temperature on the △I value, Figure S13: Effect of time on the △I value, Figure S14: Effect of Apt on the △I value, Figure S15: Effect of binding time on the △I value, Figure S16: Working curve for the SERS determination of Apt–CD-GN–AgNO_3_–TSC–BPA, Figure S17: Working curve for the SERS determination of Apt–CD_Ca_–AgNO_3_–TSC–BPA, Figure S18: Working curve for the SERS determination of Apt–CD_CN_–AgNO_3_–TSC–BPA, Figure S19: Working curve for the SERS determination of Apt–AgNP –AgNO_3_–TSC–BPA, Table S1: The catalytic effects of various catalyst and the inhibiting effect of Apt, Table S2: Selectivity of the analysis of BPA by the SERS method, Table S3: Sample analysis results (*n* = 5).
