# Supplementary material for: Aptamer-Adjusted Carbon Dot Catalysis-Silver Nanosol SERS Spectrometry for Bisphenol A Detection
Source: Nanomaterials (Basel). 2022 Apr 17;12(8):1374. doi: 10.3390/nano12081374 (PMC9032719; doi:10.3390/nano12081374)
Supplement: Supplementary file 1 [file nanomaterials-12-01374-s001.zip › nanomaterials-1657645-supplementary.pdf]

Supplementary Materials

Aptamer-Adjusted Carbon Dot Catalysis-Silver Nanosol SERS Spectrometry for Bisphenol A Detection

Yuqi Xie [1], Lu Ma [1], Shaoming Ling [1], Huixiang Ouyang [1,2,*], Aihui Liang [2] and Zhiliang Jiang [2,*]

1 Key Laboratory of Regional Ecological Environment Analysis and Pollution Control in Western Guangxi (Baise University), Education Department of Guangxi Zhuang Autonomous Region, College of Chemistry and Environment Engineering, Baise University, Baise 533000, China; xiekey@sina.com (Y.X.); malulu2022@163.com (L.M.); lingshaoming@sohu.com (S.L.)

2 Guangxi Key Laboratory of Environmental Pollution Control Theory and Technology, Guangxi Normal University, Guilin 541004, China; ahliang2008@163.com

* Correspondence: huixiang73@163.com (H.O.); zljiang@mailbox.gxnu.edu.cn (Z.J.)

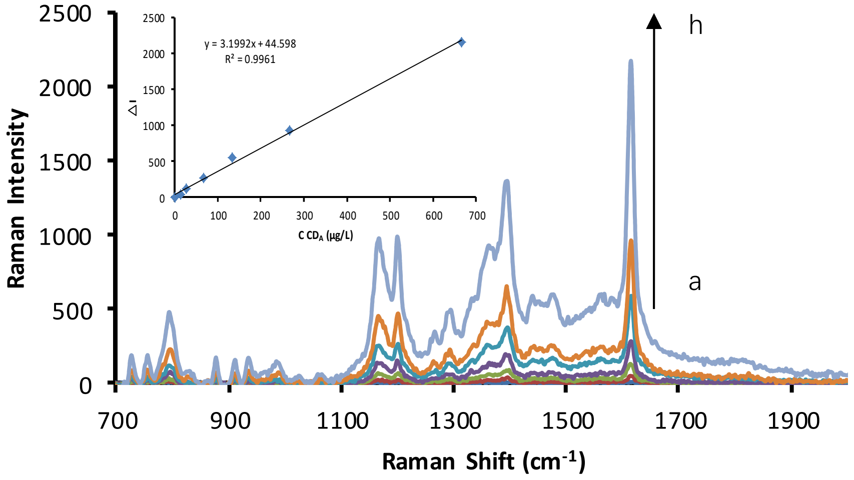

Figure S1. SERS spectra of CD-GN- $AgNO_3$-trisodium citrate (0, 13.33, 26.67, 66.67, 133.33, 266.67, 666.67 μg/L) CD-GN + 1.33 mmol/L $AgNO_3$ + 4.67 mmol/L trisodium citrate +85℃+21 min +3.33×10^{-7} mol/L VBB+0.02 mol/L NaCl

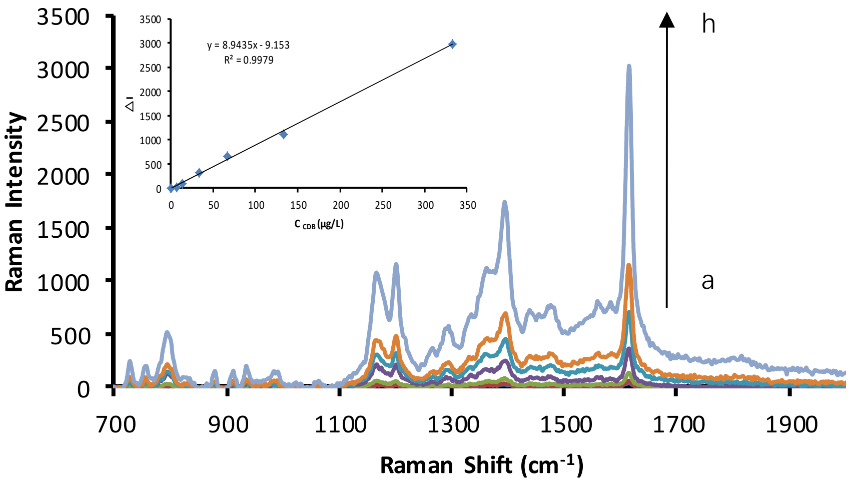

Figure S2. SERS spectra of CDCa- $AgNO_3$-trisodium citrate
(0, 6.67, 13.33, 33.33, 66.67, 133.33, 333.33 μg/L) CDCa + 1.33 mmol/L $AgNO_3$+ 4.67 mmol/L trisodium citrate +85℃+21 min +3.33×10^{-7} mol/L VBB+0.02 mol/L NaCl

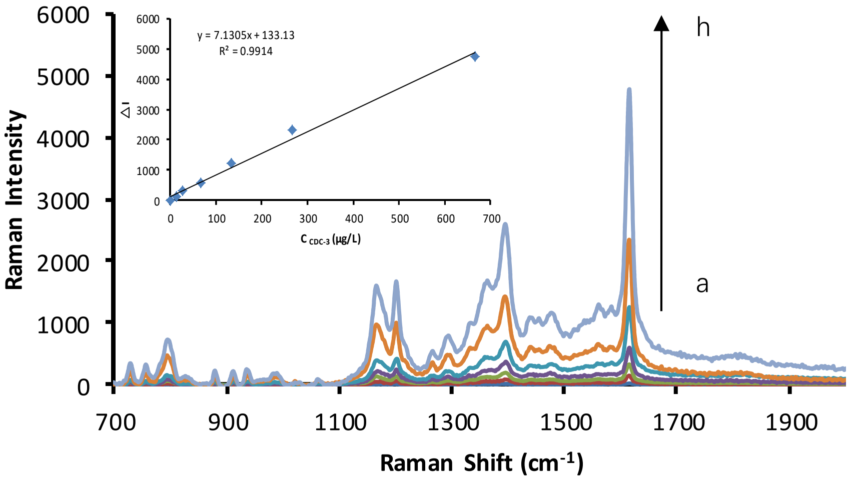

Figure S3. SERS spectra of CD-CN- $AgNO_3$-trisodium citrate
(0, 13.33, 26.67, 66.67, 133.33, 266.67, 666.67 μg/L) CD-CN2+ 1.33 mmol/L $AgNO_3$ + 4.67 mmol/L trisodium citrate +85℃+21 min +3.33×10^{-7} mol/L VBB+0.02 mol/L NaCl

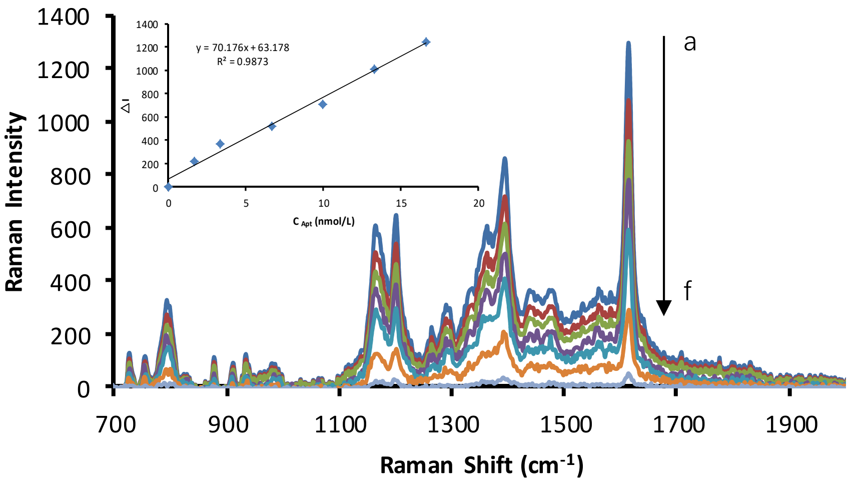

Figure S4. SERS spectra of Apt- CD-CN- $AgNO_3$-trisodium citrate

(0, 1.67, 3.33, 6.67, 10, 13.33,16.67 nmol/L) Apt + 266.67 μg/L CD-CN2 + 1.33 mmol/L $AgNO_3$+ 4.67 mmol/L trisodium citrate +85℃+21 min +3.33×10^{-7} mol/L VBB+0.02 mol/L NaCl

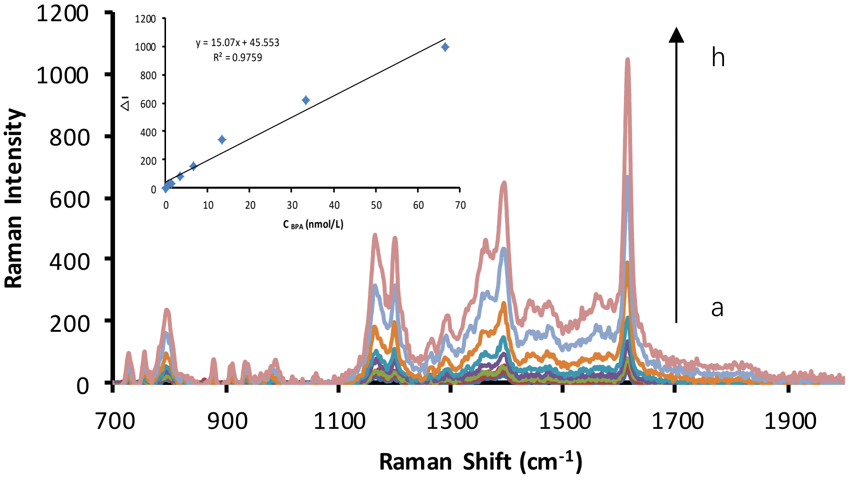

Figure S5. SERS spectra of BPA-Apt- CD-GN- $AgNO_3$-trisodium citrate

16.67 nmol/L Apt + 266.67 μg/L CD-GN + (0, 0.67, 1.33, 3.33, 6.67, 13.33, 33.33, 66.67 nmol/L) BPA + 1.33 mmol/L $AgNO_3$ + 4.67 mmol/L trisodium citrate +85℃+21 min +3.33×10^{-7} mol/L VBB+0.02 mol/L NaCl

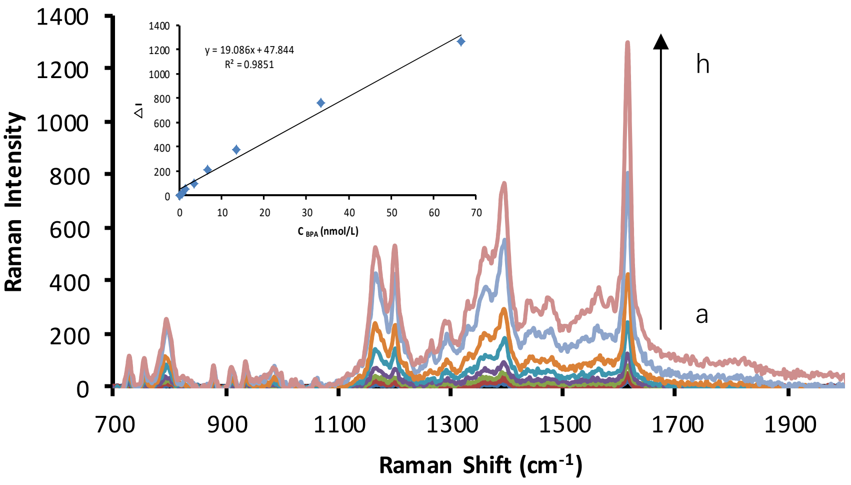

Figure S6. SERS spectra of BPA-Apt- CD_{Ca}- $AgNO_3$-trisodium citrate
13.33 nmol/L Apt + 133.33 μg/L CD_{Ca} + (0, 0.67, 1.33, 3.33, 6.67, 13.33, 33.33, 66.67 nmol/L) BPA + 1.33 mmol/L $AgNO_3$ + 4.67 mmol/L trisodium citrate +85℃+21 min +3.33×10^{-7} mol/L VBB+0.02 mol/L NaCl

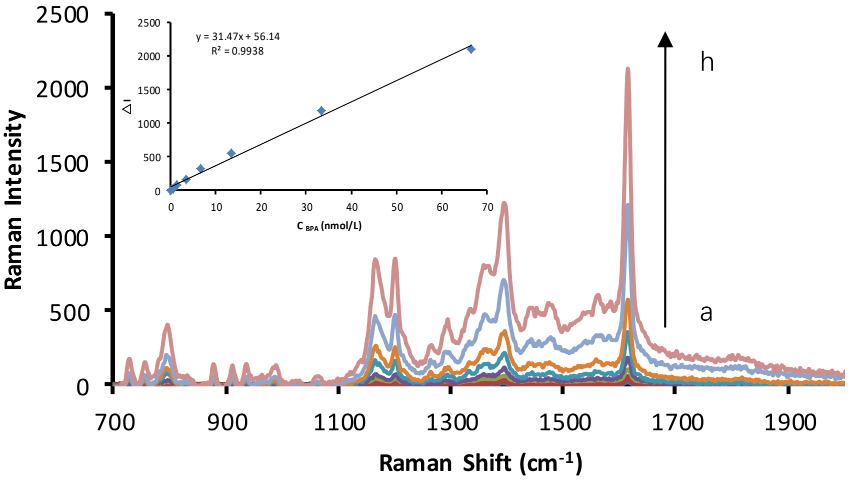

Figure S7. SERS spectra of BPA-Apt- CD-CN- $AgNO_3$-trisodium citrate
13.33 nmol/L Apt + 266.67 μg/L CD-CN2 + 0.67-66.67 nmol/L BPA + 1.33 mmol/L $AgNO_3$ + 4.67 mmol/L trisodium citrate +85℃+21 min +3.33×10^{-7} mol/L VBB+0.02 mol/L NaCl

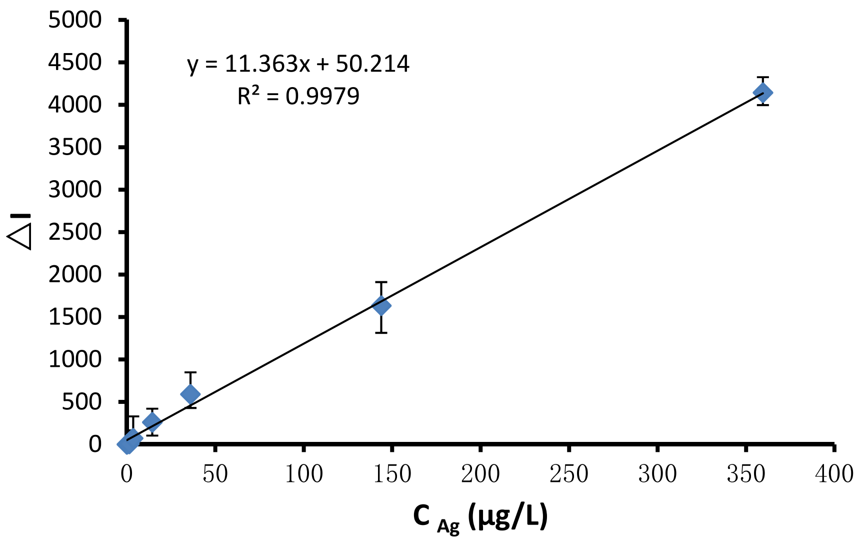

Figure S8. The effect of AgNP on SERS intensity

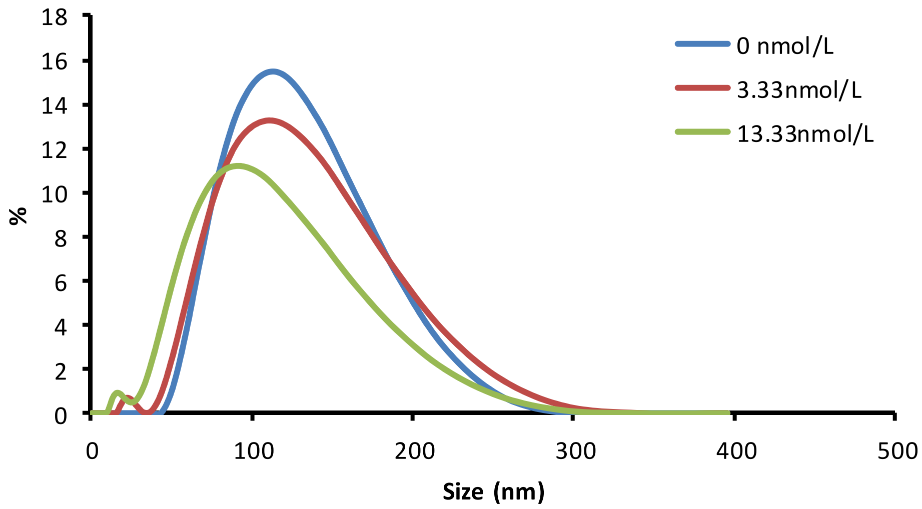

Figure S9. Laser scattering image of Apt- CD-FN3 - $AgNO_3$-TSC-BPA system 20.67 nmol/L Apt + 333.33 μg/L CD-FN3 + 1.33 mmol/L $AgNO_3$ + 4.67 mmol/L TSC+85℃+21 min；b. a+ 3.33 nmol/L BPA； c. a+ 3.33 nmol/L BPA

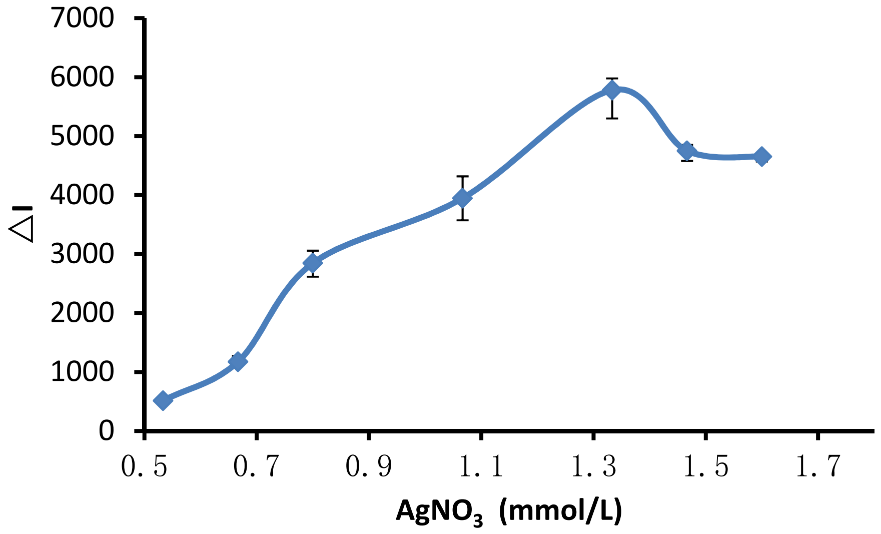

Figure S10. Effect of $AgNO_3$ concentration on the △I value
333.33 μg/L CD-FN3 +x mmol/L $AgNO_3$+6.67 mmol/L TSC+85℃+ 21 min +3.33×10^{-7} mol/L VBB+0.02 mol/L NaCl

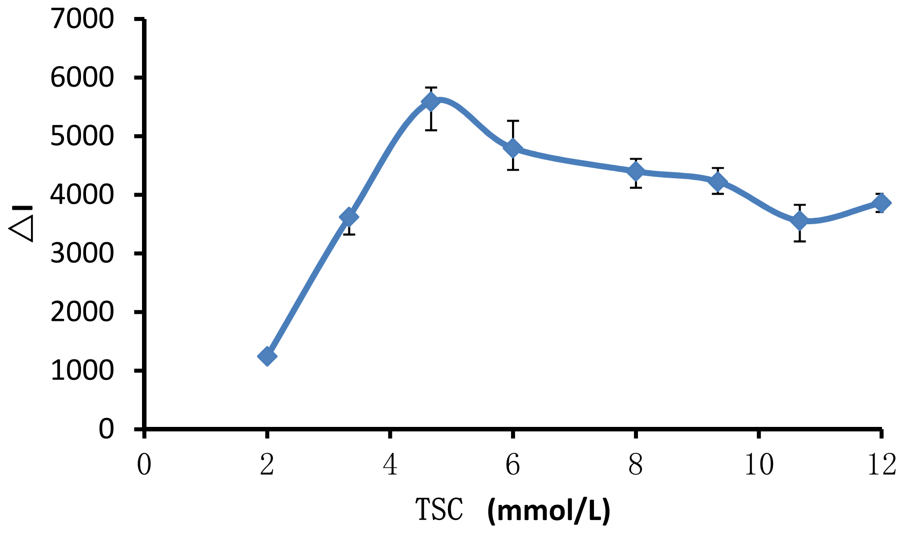

Figure S11. Effect of TSC concentration on the △I value
333.33 μg/L CD-FN3 + 1.33 mmol/L $AgNO_3$+x mmol/L TSC+85℃+ 21 min +3.33×10^{-7} mol/L VBB+0.02 mol/L NaCl

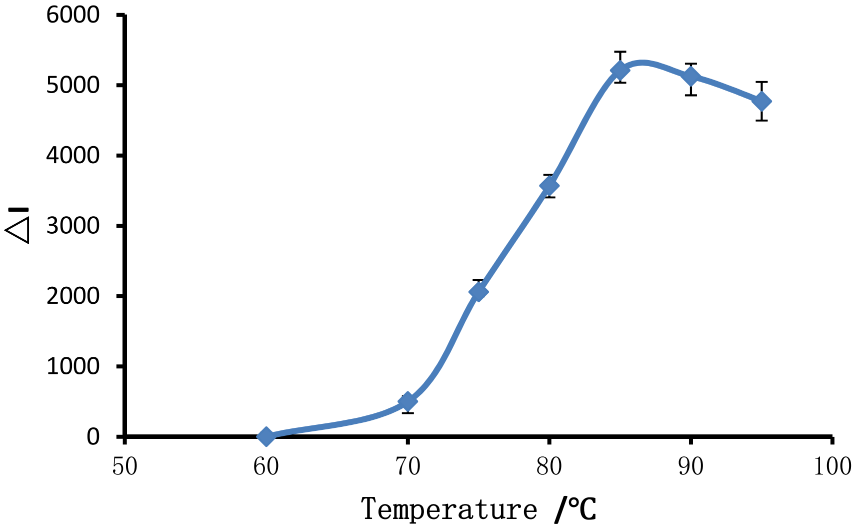

Figure S12. Effect of temperature on the △I value

333.33 μg/L CD-FN3 + 1.33 mmol/L $AgNO_3$+4.67 mmol/L TSC+ x ℃+ 21 min +3.33×10^{-7} mol/L VBB+0.02 mol/L NaCl

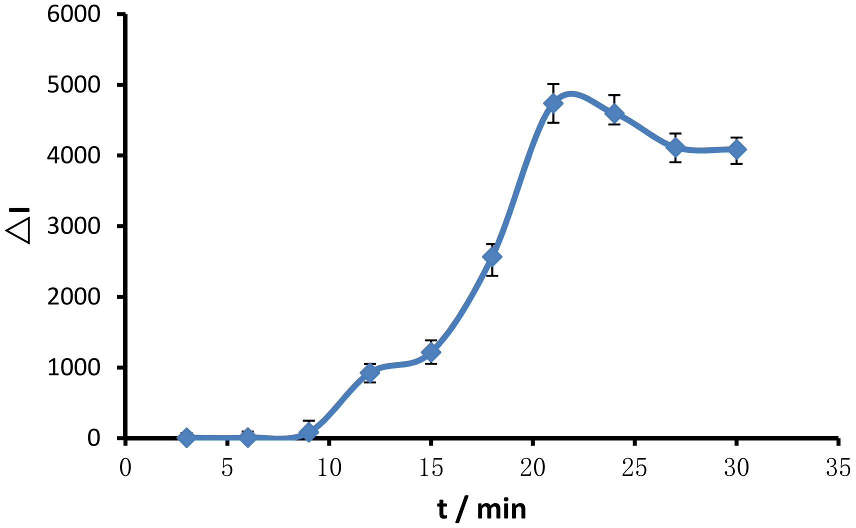

Figure S13. Effect of time on the △I value

333.33 μg/L CD-FN3 + 1.33 mmol/L $AgNO_3$+ 4.67 mmol/L TSC+85℃+ x min +3.33×10^{-7} mol/L VBB+0.02 mol/L NaCl

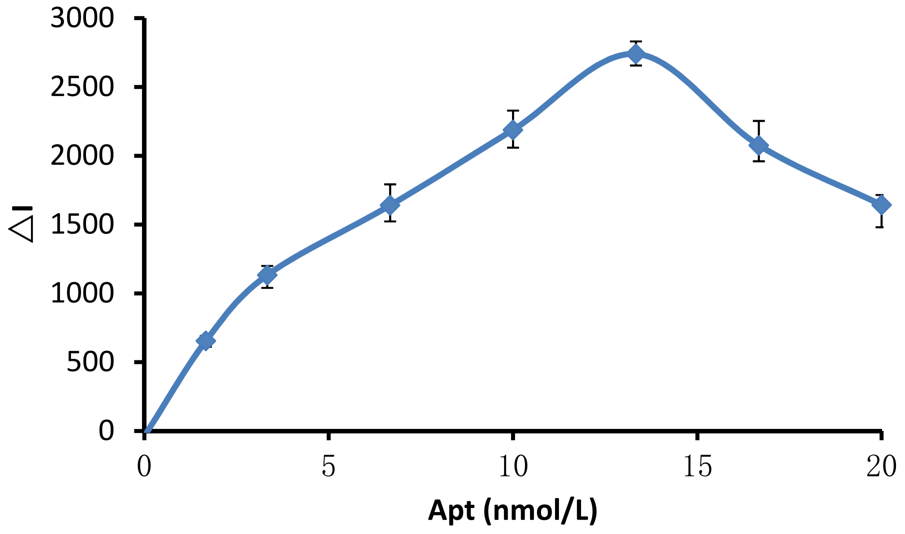

Figure S14. Effect of Apt on the △I value
x nmol/L Apt + 5 μg/L BPA+333.33 μg/L CD-FN3 + 1.33 mmol/L $AgNO_3$+ 4.67 mmol/L TSC+85℃+ 21 min +3.33×10^{-7} mol/L VBB+0.02 mol/L NaCl

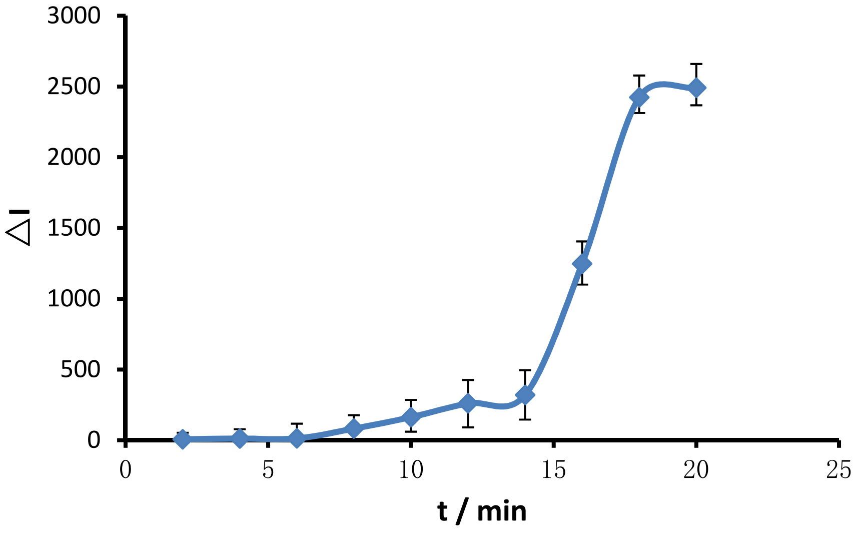

Figure S15. Effect of binding time on the △I value
13.33 nmol/L Apt + 5 μg/LBPA+333.33 μg/L CD-FN3 + 1.33 mmol/L $AgNO_3$+ 4.67 mmol/L TSC+85℃+ 21 min +3.33×10^{-7} mol/L VBB+0.02 mol/L NaCl

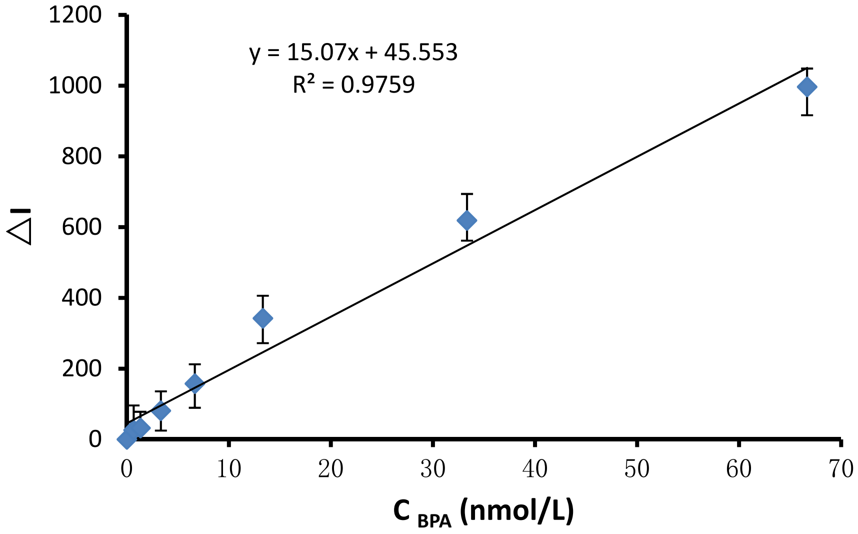

Figure S16. Working curve for the SERS determination of Apt-CD-GN-$AgNO_3$-TSC-BPA 16.67 nmol/L Apt + 266.67 μg/L CD-GN + 0.67-66.67 nmol/L BPA + 1.33 mmol/L $AgNO_3$+ 4.67 mmol/L TSC+85℃+21 min +3.33×10^{-7} mol/L VBB+0.02 mol/L NaCl

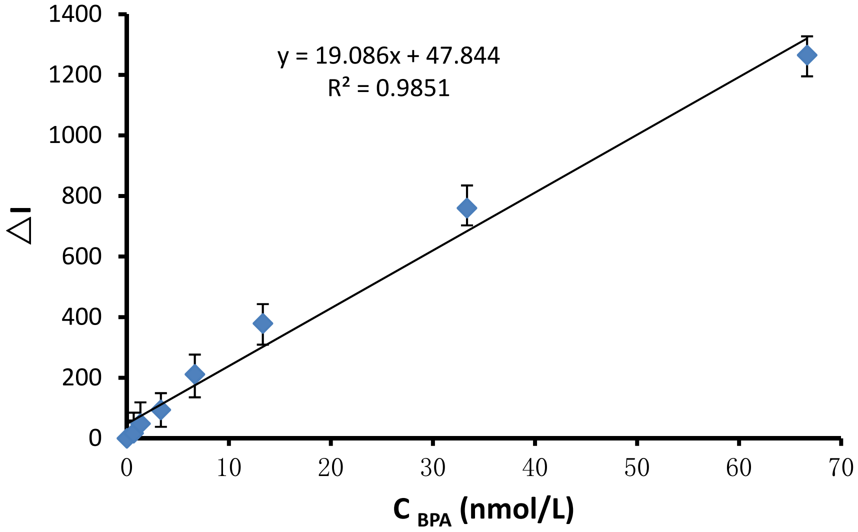

Figure S17. Working curve for the SERS determination of Apt-CD_{Ca}-$AgNO_3$-TSC-BPA 13.33 nmol/L Apt + 133.33 μg/L CD_{Ca} + 0.67-66.67 nmol/L BPA + 1.33 mmol/L $AgNO_3$+ 4.67 mmol/L TSC+85℃+21 min +3.33×10^{-7} mol/L VBB+0.02 mol/L NaCl

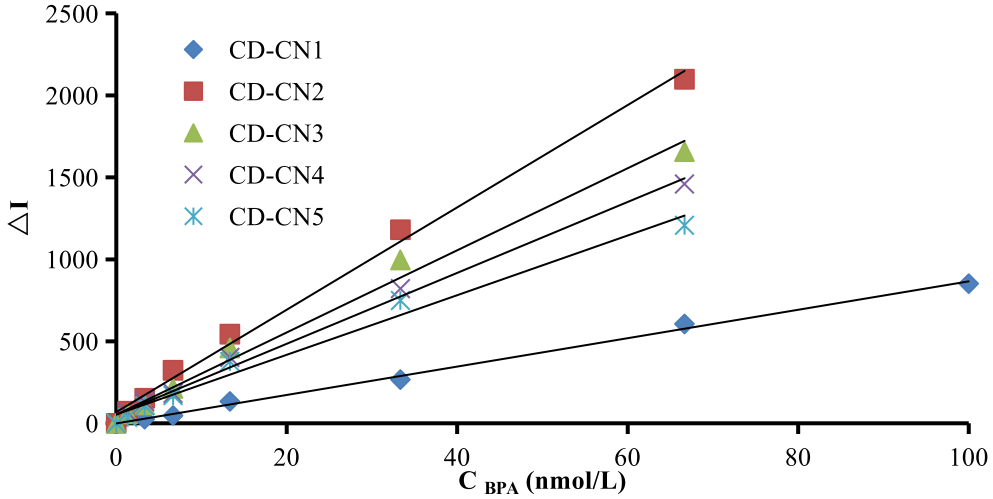

Figure S18. Working curve for the SERS determination of Apt-CD$_{CN}$-AgNO$_3$-TSC-BPA 13.33 nmol/L Apt + CD$_{CN}$ + 3.33-100 nmol/L BPA + 1.33 mmol/L AgNO$_3$+ 4.67 mmol/L TSC+85℃+21 min +3.33×10^{-7} mol/L VBB+0.02 mol/L NaCl

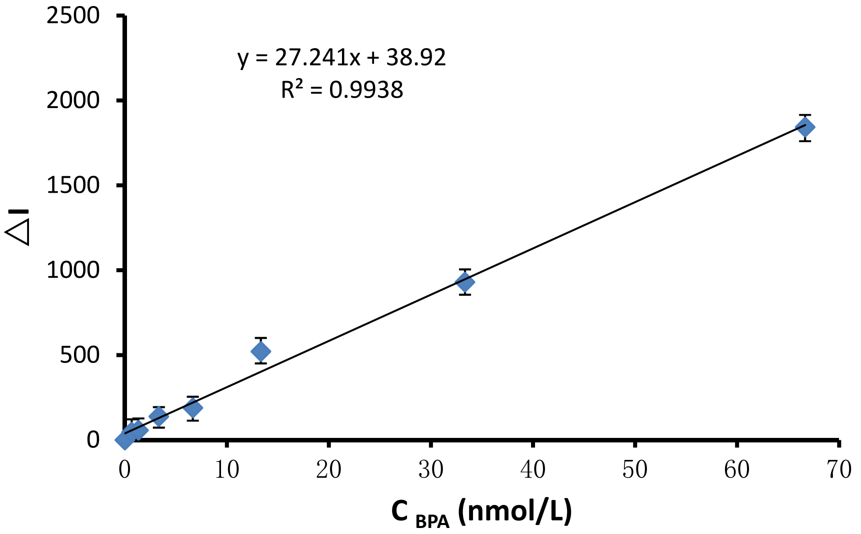

Figure S19. Working curve for the SERS determination of Apt- AgNP -AgNO$_3$-TSC-BPA 6.67 nmol/L Apt + 143.82 μg/L AgNP + 0.67-66.67 nmol/L BPA + 1.33 mmol/L AgNO$_3$+ 4.67 mmol/L TSC+85℃+21 min +3.33×10^{-7} mol/L VBB+0.02 mol/L NaCl

Table S1. The catalytic effect of various catalyst and the inhibiting effect of Apt

Nanocatalyst	Regression equation	Linear range	Correlation coefficient (R^2)
CD-FN0	$\triangle I_{1614cm-1} = 1.00\ C + 66.06$	8.33~1666.67 μg/L	0.985
CD-FN1	$\triangle I_{1614cm-1} = 5.14\ C + 89.51$	3.33~833.33 μg/L	0.9848
CD-FN2	$\triangle I_{1614cm-1} = 14.00\ C + 12.25$	1.67~333.33 μg/L	0.9995
CD-FN3	$\triangle I_{1614cm-1} = 18.97\ C + 15.22$	1.67~333.33 μg/L	0.9985
CD-FN4	$\triangle I_{1614cm-1} = 13.94\ C + 62.59$	1.67~333.33 μg/L	0.989
CD-FN5	$\triangle I_{1614cm-1} = 6.71\ C + 122.74$	8.33~833.33 μg/L	0.994
CD-SN0	$\triangle I_{1614cm-1} = 0.28\ C + 61.53$	83.33~8333.33 μg/L	0.9793
CD-SN1	$\triangle I_{1614cm-1} = 3.41\ C + 73.30$	8.33~1666.67 μg/L	0.9982
CD-SN2	$\triangle I_{1614cm-1} = 7.16\ C + 207.03$	3.33~833.33 μg/L	0.9772
CD-SN3	$\triangle I_{1614cm-1} = 2.48\ C + 112.88$	16.67~1666.67 μg/L	0.9702
CD-SN4	$\triangle I_{1614cm-1} = 2.52\ C + 86.61$	8.33~1666.67 μg/L	0.9889
CD-SN5	$\triangle I_{1614cm-1} = 2.29\ C + 97.20$	8.33~1666.67 μg/L	0.9627
CD_{GN}	$\triangle I_{1614cm-1} = 3.2\ C + 44.60$	13.33~666.67 μg/L	0.9961
CD_{Ca}	$\triangle I_{1614cm-1} = 8.94\ C - 9.15$	6.67~333.33 μg/L	0.9979
CD-CN1	$\triangle I_{1614cm-1} = 0.39\ C + 36.46$	70~2800 μg/L	0.9873
CD-CN2	$\triangle I_{1614cm-1} = 7.13\ C + 133.13$	13.33~666.67 μg/L	0.9914
CD-CN3	$\triangle I_{1614cm-1} = 5.44\ C - 23.67$	13.33~666.67 μg/L	0.9989
CD-CN4	$\triangle I_{1614cm-1} = 6.30\ C + 136.53$	13.33~666.67 μg/L	0.9903
CD-CN5	$\triangle I_{1614cm-1} = 1.77\ C + 49.77$	13.33~1333.33 μg/L	0.9946
Apt- CN3	$\triangle I_{1614cm-1} = 70.17\ C + 63.18$	1.67~16.67 nmol/L	0.9873
Apt- CD-FN3	$\triangle I_{1614cm-1} = 387.19\ C + 213.08$	0.33~10 nmol/L	0.9249
Apt- CD-SN2	$\triangle I_{1614cm-1} = 608.81\ C + 154.77$	0.33~6.67 nmol/L	0.9784
AgNP	$\triangle I_{1614cm-1} = 11.36\ C + 50.21$	1.44~359.56 μg/L	0.9979

Table S2. Selectivity of the analysis of BPA by the SERS method

coexistent ion	Limit times	coexistent ion	Limit times	coexistent ion	Limit times
CO_3^{2-}	1200	K^+	800	Ethyl alcohol	800
Na^+	1000	Zn^{2+}	500	Acetone	800
HCO_3^-	600	Mg^{2+}	200	Phenol	100
Cl^-	1000	NO_3^-	800	HSA	10
Br^-	200	Ca^{2+}	200	BSA	10
BrO_3^-	100	Cu^{2+}	80	Glycine	100
F^-	100	NH_4^+	400	Phenylalanine	50
Ba^{2+}	400	Al^{3+}	100	Valine	50
Fe^{2+}	100	Fe^{3+}	100	Tryptophan	40
Ni^{2+}	80			Glutamic acid	50

Table S3. Sample analysis results (n=5)

Sample	Found		Added	Determination value	Recovery/%
Plastic film 1	-	-	3.33 nmol/L	3.32 nmol/L	99.7%
Plastic film 2	-	-	3.33 nmol/L	3.36 nmol/L	100.9%
Polythene bag 1	-	-	3.33 nmol/L	3.40 nmol/L	102.1%
Polythene bag 2	-	-	3.33 nmol/L	3.28 nmol/L	98.5%
unbranded grocery bag	2.5 nmol/L	2.85 mg/g	3.33 nmol/L	6.01 nmol/L	105.4%
Plastic drinking cup 1	1.4 nmol/L	1.59 mg/g	3.33 nmol/L	4.72 nmol/L	99.7%
Plastic drinking cup 2	-	-	3.33 nmol/L	3.41 nmol/L	102.4%
